# Preprocessing Pipelines including Block-Matching Convolutional Neural Network for Image Denoising to Robustify Deep Reidentification against Evasion Attacks

**DOI:** 10.3390/e23101304

**Published:** 2021-10-03

**Authors:** Marek Pawlicki, Ryszard S. Choraś

**Affiliations:** 1 ITTI Sp. z o.o., 61-612 Poznań, Poland; 2 Institute of Telecommunications and Computer Science, Bydgoszcz University of Science and Technology, 85-796 Bydgoszcz, Poland; Ryszard.Choras@utp.edu.pl

**Keywords:** deep learning, computer vision, adversarial attacks, adversarial defences

## Abstract

Artificial neural networks have become the go-to solution for computer vision tasks, including problems of the security domain. One such example comes in the form of reidentification, where deep learning can be part of the surveillance pipeline. The use case necessitates considering an adversarial setting—and neural networks have been shown to be vulnerable to a range of attacks. In this paper, the preprocessing defences against adversarial attacks are evaluated, including block-matching convolutional neural network for image denoising used as an adversarial defence. The benefit of using preprocessing defences comes from the fact that it does not require the effort of retraining the classifier, which, in computer vision problems, is a computationally heavy task. The defences are tested in a real-life-like scenario of using a pre-trained, widely available neural network architecture adapted to a specific task with the use of transfer learning. Multiple preprocessing pipelines are tested and the results are promising.

## 1. Introduction

Artificial neural networks offer a collection of benefits which have proved useful in image processing, especially in tasks including artificial-intelligence-based computer aided detection [1]. The progress of the last decade allowed to push the results obtained by artificial neural networks to levels surpassing human performance, in select tasks [2]. In computer vision, deep neural networks became the go-to solution for a wide variety of problems [3], capable of producing an impressive result in a sensible time frame [4]. Recently, artificial neural networks found success in person reidentification [5,6,7].

In general, reidentification (reID) refers to the process of re-attaching publicly available data to an anonymised record in order to discover the identity of an individual. In the context of computer vision, the phrase refers to the ability of an image recognition system to spot an individual across different cameras, and different angles [7]. ReID is a challenging task which stirred up a significant amount of research recently, particularly due to the significant benefits it could bring for public safety [7]. The use in the context of intelligent surveillance systems forces the consideration of adversarial behaviour against the artificial intelligence (AI) technologies used for reID. In a real-world scenario, impressive detection metrics are not the only thing that matters [8]. The current trend in reID involves the use of deep neural networks, which have been proven to be susceptible to a novel kind of attacks [9,10,11,12].

Deep neural networks, particularly convolutional neural networks (CNN), are widely used for the CV tasks [13]; some of the best-performing ImageNet contest architectures were based on the premise of utilising convolutional layers. The network architectures tend to be very deep: Inception features over 6 million trainable parameters [14,15], ResNet18 (Residual neural network) over 11 million [16], AlexNet over 60 million [17], VGG16 (Visual Geometry Group) over 138 million [18], etc. Therefore, training a top-tier deep neural network is a huge computational endeavour [19]. In order not to repeat this effort for each task, transfer learning can be employed [20]. Transfer learning leverages pre-trained networks, essentially using them as feature extractors with frozen weights, feeding samples to the network and only training the added dense layers at the output end of the topology. However, the use of openly-available, pre-trained networks poses a security problem in an adversarial setting, as it raises the capability of the attacker [21,22].

The idea of attacking deep neural networks has focused the attention of the deep learning community over the last few years [23,24,25]. A range of adversarial attacks effective against AI were discovered, uncovering the vulnerabilities of data driven technologies [25]. In this work, the attacks performed at test-time are considered, which are known as evasion attacks [26,27].

The goal of an Evasion attack is to force the AI-based system to misclassify a particular sample. This is achieved by adding a specifically crafted noise to the tested sample. This added noise, in case of images, is imperceptible to humans, but leverages the ’intriguing properties of neural networks’ to fool the AI algorithm [28]. The issue of defending against those attacks is a fierce arms race and the satisfactory defence has not yet emerged [29].

The algorithms and technologies presented in this paper were used to form a submission to the reidentification defences track of the H2020 SPARTA SAFAIR contest. The task was formulated around the CelebA face recognition dataset [30,31]. The dataset, as used in the task, featured 5304 classes, with 85,612 samples in the training subset and 28,523 samples in the testing set. The objective of the defensive track was to propose ways of preventing adversarial samples from lowering the accuracy of the face recognition model. The following sections describe the specific technologies used for defining the submission of the contest, the rationale behind those choices, the formulated defences, and provide the results of the experiments.

As such, the research and, thus, the paper is conducted and formulated to answer the following research question:

- **RQ1** Is it possible to use data preprocessing methods to robustify an ANN-based classifier against adversarial evasion attack in computer vision (CV)?
- **RQ2** Does using all the identified defensive preprocessing methods provide a better protection than using just a selection of those?

Thus, the innovative contribution of this paper comes in the formulation and evaluation of a plug-and-play preprocessing pipeline for robustification of already-existing or pre-trained CV classifiers, easily deployable in a real-world situation and saving on the cost of re-training the classifier

The paper is structured as follows: In Section 2, the related works are introduced and the most important categories of defences are described. Section 3 lists the setup of the used reidentification pipeline, showcases the effects of the adversarial attacks and introduces the specific defences, including the block-matching convolutional neural network (BMCNN) for image denoising, which, to the best of our knowledge, has never before been used to counter adversarial attacks. Section 4 contains the experimental setups and the results obtained by specific pipelines. Section 5 encompasses the conclusions along with the impact the defensive pipeline has over a clean dataset.

## 2. Related Works

The advent of adversarial perturbations revealed the vulnerabilities of contemporary AI-based technologies. There is a considerable body of research into both the attacks and the defences. However, as noted by [32], the construction of a theoretical model of crafting adversarial perturbations is problematic, as it is a sophisticated optimisation procedure for most machine learning models. This absence of a theoretical baseline makes it troublesome to verify whether administering a certain defence can proof a system against a certain set of attacks. This situation finds its expression in the fact that whenever a new defence is proposed, a new attack capable of breaking through that defence appears [33,34,35,36,37,38].

Against this canvas, the authors of [38] propose a set of guidelines for research into the defensive mechanisms against adversarial attacks, listing common pitfalls and a range of best practices. There is a substantial body of work gathering both the available attacks and possible defences geared towards machine and deep learning [27,29,32,39,40,41,42] and even specifically deep learning in computer vision [23,43,44].

A thorough analysis of the sources allows one to roughly divide the adversarial defences into these categories:

1. Gradient masking;
2. Input reconstruction;
3. Detectors.

According to [29], the category of gradient masking encompasses defences which fit either intentionally or unintentionally. This category of defences relies on making the gradient unfit for the operation of the attack algorithms. Some defences do not aim at gradient masking specifically, but achieve it as a by-product of defensive procedures. One of the most popular approaches, adversarial training, frequently has a gradient masking effect, even though it is not the goal of the process.

Adversarial (re)training is considered as the brute-force approach [32]. The procedure relies on crafting adversarial samples and including them in the training set. The problem with retraining the whole classifier is the computational cost of such course of conduct. This problem will be touched upon later in this paper.

The defences in the input reconstruction category perform various forms of input pre-processing. Although it might be possible to circumvent those methods in a scenario where the attacker has full knowledge of the system, in a real-world setting the defences from this category can be very effective, and computationally much cheaper in use than retraining. The detection approaches are effective as long as the adversary is not aware of the existence of the detector. For an attacker of sufficient capability it is possible to build an adversarial sample which, at the same time, circumvents the detector and fools the classifier, as proven by [33].

## 3. Materials and Methods

### 3.1. Classifier Setup

In this work, the VGG-face network was used [45] with the pre-trained ‘resnet-50’ [46] architecture. VGGFace is trained on a dataset containing 2.6 million face images of over 2.6k people. The resnet50 network is a CNN assembled of 50 layers. The detailed hyperparameter setup of the entire network can be found in [45]. The final layer of the pre-trained network is AveragePooling2D with the shape of (None, 1, 1, 2048). To perform transfer learning, a dense layer of 2048 neurons is added to the the pre-trained network, followed by a dropout layer, and wrapped up by the softmax layer set with the number of neurons equal to the number of classes. The added dense layer uses the rectified linear unit (ReLU) activation function. The weights between the AveragePooling layer and the dense layer along with the weights between the dense layer and the output layer constitute the part of the network that is trained on the CelebA dataset, with the weights of the remainder of the network frozen. The batch size used for training was set to 1, while early stopping was used to find the optimal number of epochs, which capped at 32. Multiple different hyperparameters setup were tested, and learning rate scheduling was also tested. For the reduced dataset used in the experiments the default learning rate of straight 0.01 proved optimal.

The trainable part of the model contains 15,064,248 parameters when it is prepared to recognise all the 5304 identities found in the CelebA dataset. To allow fast prototyping, a toy model was built on fourteen most populated classes in the CelebA dataset. The most populated classes were chosen to avoid having to deal with the data imbalance problem, allowing the research to focus on adversarial defences. Changing just the number of classes allowed to reduce the number of trainable parameters to just over 4 million; a reduction of over 70%. The prior probability of the occurrence of each of the classes is displayed in Table 1.

Multi-task cascaded convolutional neural networks (MTCNN) is a technique capable of spotting faces and extracting them for later processing by other networks. A state-of-the-art face recognition processing pipeline consists of MTCNN for face detection and landmark placement, and a CNN used for placing the extracted face in adequate categories [47,48,49]. In this work, MTCNN is used for preprocessing the CelebA images for both training and testing. The CelebA subset selected for the formulation of the model was further split into the training set and the testing set. The classifier performance on the test set containing the 14 most populated classes is presented in Table 2.

For better evaluation of the effects of adversarial perturbations and adversarial defences, the misclassified samples were removed from the set, manually pushing the performance to 100% accuracy. That way, any adversarial perturbations are registered as drops in performance, avoiding a situation where an attack pushes the misclassified sample to the correct class. Furthermore, the way the defences affect the classifier performance is more clearly readable.

### 3.2. Adversarial Attacks

**Figure 1:**
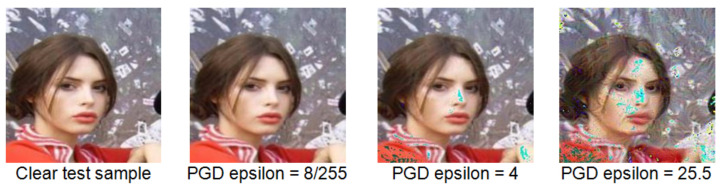
The effects of different strengths of the attacks on the image.

The testing set was then subjected to the procedure of creating the adversarial samples. To produce the adversarial attacks, the projected gradient descent (PGD) method was used, considering PGD as the universal first-order adversary, following [50]. The maximum number of iterations was set to 100, the epsilon step to 0.1. The value of epsilon determines the maximum size of perturbation allowed for the attack. Along with the number of iterations, multiple values of epsilon were tested to simulate different strengths of attack. The effect different strengths of the attacks have on the image can be seen in Figure 1. The pictures are reformatted to fit the vgg-face input shape. The effects of PGD eps = 4 on the performance of the classifier can be seen in Table 3.

### 3.3. Defences

There have been a number of defences proposed by the research community [51]. The task is to design robust AI tools that are resilient to adversarial attacks. Some methods rely on retraining the entire classifier using attacks generated with the known attack methods [52]. This method, called adversarial training, not only impacts the effectiveness of the classifier, but also requires an immense computational effort. The proposition contained in this section utilises the idea of using pre-processing methods to robustify existing AI-based classifiers, so as the users do not need to re-train their models. The proposed methods are accompanied by an assessment of how the defensive measures affect the classifier performance, which helps optimise the resiliency of AI against the loss of performance some defences introduce.

#### 3.3.1. JPEG Compression

**Figure 2:**
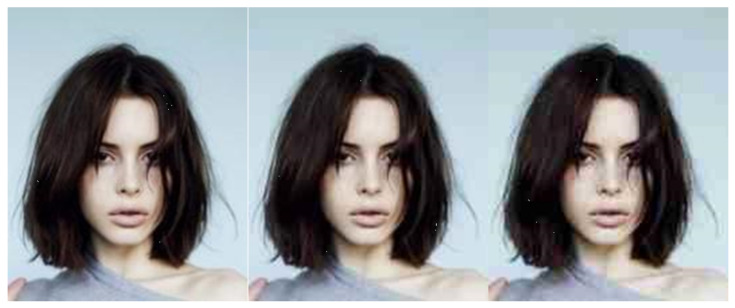
JPEG compression.

The Joint Photographic Experts Group (JPEG) compression used as adversarial defence relies on the fact that JPEG-compressed images are very prevalent in contemporary usage. Following the authors of [53], who noted that JPEG compression often has the ability to reverse the effects of small adversarial perturbations, the technique is evaluated here for the use as a purely pre-processing defence against adversarial attacks. The compression has the effect of removing additive artefacts in square blocks of an image, effectively working as a filter removing adversarial perturbations [54]. The effect of different magnitudes of compression (20, 40, 80) can be seen in Figure 2. The results of the classifier using JPEG compression with quality set to 20 on PGD attacks with epsilon = 4 can be found in Table 4.

#### 3.3.2. Gaussian Data Augmentation

**Figure 3:**
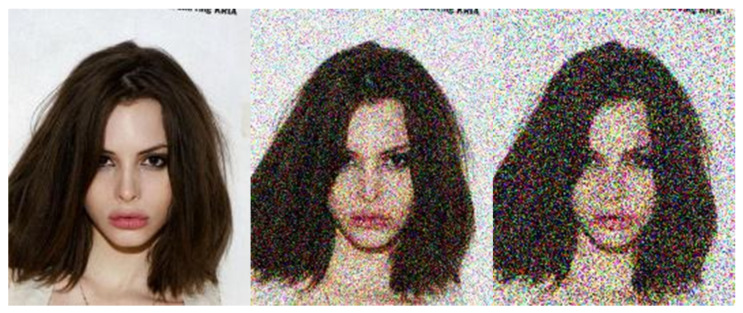
Gaussian augmentation—sigma 255.0/5, 255.0/17, 255.0/3.

Gaussian data augmentation [55] is a process of adding Gaussian noise to a sample. This method is proven not to produce adversarial samples and can reverse the effects of known adversarial attacks. Image samples with different sigma settings can be seen in Figure 3. The value of sigma expresses the variance.

#### 3.3.3. Local Spatial Smoothing

**Figure 4:**
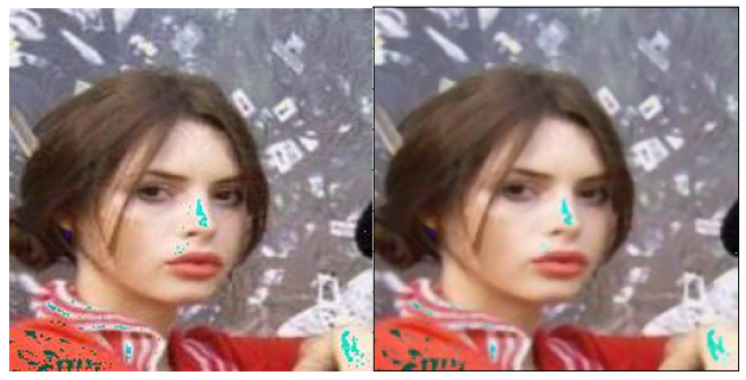
The image before and after spatial smoothing.

Following the research of [56], spatial smoothing can be used to reduce the effects of added adversarial noise. The algorithm uses local blurring filters to remove the effects of adversarial noise. The approach is one of the feature squeezing methods and can be effectively applied as a pre-processor-based defence. The image before and after spatial smoothing can be seen in Figure 4.

#### 3.3.4. Total Variance Minimisation

**Figure 5:**
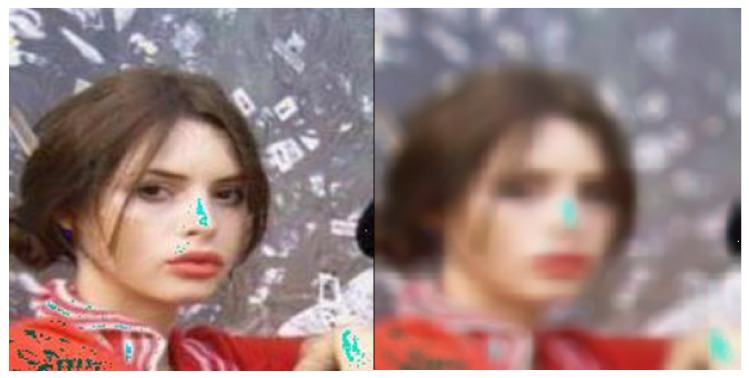
The image before and after total variance minimisation.

Total variance minimisation is a model-agnostic preprocessor approach. In the original paper [57], the defence is used for retraining the model and then the inputs are also pre-processed at test time. The method reassembles the image by rebuilding a randomly chosen set of pixels with the plainest depiction of these pixels. The image before and after total variance minimisation can be seen in Figure 5.

#### 3.3.5. Block-Matching Convolutional Neural Network (BMCNN) for Image Denoising as an Adversarial Defence

Following the work in image denoising presented in [58], and extending the idea of applying autoencoders as adversarial defences [59], the BMCNN is proposed for the a method of robustifying the image recognition system against adversarial attacks. BMCNN is an attempt to merge two leading approaches to image denoising: non-local self-similarity prior based methods [60] and feed-forward denoising with the use of convolutional neural networks [61]. The method is applied as a pre-processor to remove adversarial noise before the sample is fed to the classifier. The results of the BMCNN with sigma set to 20 used on adversarial samples created with PGD with epsilon set to four can be seen in Table 5. The value of sigma has been chosen experimentally.

## 4. Results

**Figure 6:**
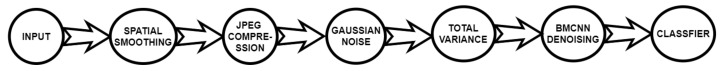
A defensive pipeline which utilises all the researched defences.

The low computational cost of the preprocessors in comparison with re-training the classifier allows to mix and match the defences. The experiments show that some pipelines are more effective than others. An example of a defensive pipeline which utilises all the researched defences is displayed in Figure 6.

The pipeline makes intuitive sense, as blurring the image should remove some of the artefacts added by PGD, same for JPEG compression, then adding Gaussian noise and removing it with BMCNN denoising has the potential of removing both the Gaussian and the adversarial noise at the same time. The results of this particular pipeline are shown in Table 6.

**Figure 7:**
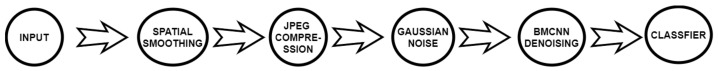
A defensive pipeline which utilises all the researched defences, except total variance minimisation.

As showcased by the results of the experiment in Table 6, the mix of defences improved the detection metrics as compared to the undefended model; however it did not perform as well as, for example, BMCNN denoising alone (Table 5). For the next experiment, the total variance minimisation preprocessor was removed, as it has a similar filtering effect as localised spatial smoothing. The pipeline is shown in Figure 7. The results of the experiment are contained in Table 7.

**Figure 8:**
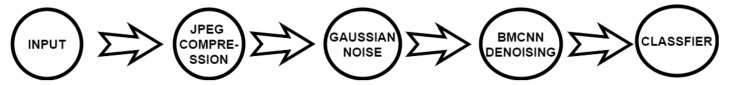
A defensive pipeline with JPEG compression, Gaussian augmentation, and BMCNN.

To find the optimal mix of preprocessors that would minimise or eliminate the effect of adversarial perturbations without significantly deteriorating the classifier results, a range of experiments was performed. The results of some of those tests are contained in Table 8 and Table 9. To assess the results of the preprocessing defences, the best performing preprocessing pipeline was tested on a clean, unperturbed set. The results of this experiment can be found in Table 10. The best performing pipeline is illustrated in Figure 8.

## 5. Conclusions

The classifier performance indicates that using preprocessing defences causes a drop in the measured metrics; at the same time, the achieved robustness is considerable. The results of the experiments prove that input transformations are an effective weapon against adversarial attacks, though the robustness comes at a cost. The utility of the proposed preprocessing pipeline solution comes in the fact that it can be used as a plug-and-play quick-fix, granting a measure of robustness against adversarial attacks without having to incur the costs of re-training the classifier. This answers RQ1 affirmatively, using preprocessing defensive methods is feasible for robustification of ANN-based classifiers against adversarial evasion attacks in computer vision tasks. The results of the experiments also point out that passing the images through a series of filters can have adverse effects on the accuracy of protected classifier. Joining all the researched preprocessing methods in one pipeline did alleviate some of the effects of the adversarial attacks. However, the accumulative distortion introduced by those methods hindered the effectiveness of the classifier to a considerable extent. Extensive experimentation made it possible to answer RQ2—some combinations are more effective than others and more effective than using all the preprocessors together.

Additionally, data augmentation is a booming area of research [62], and mixing preprocessing adversarial defences with novel approaches to data augmentation could potentially offset the performance loss of the researched defensive techniques, an approach which is part of future research.

## Figures and Tables

**Table 1:** The priors of the classes.

Class	1757	2114	2820	3227	3699	3745	3782	4262
Prior Probability (%)	6.72	6.72	7.84	7.84	7.56	7.56	7.84	6.72
	4740	4978	6568	8968	9152	9256		
Prior Probability (%)	6.72	6.72	6.72	7.00	7.00	7.00		

**Table 2:** Classifier performance on the test set containing the 14 most populated classes.

Label	Precision	Recall	f1-Score	
1757.0	1.00	1.00	1.00
2114.0	1.00	1.00	1.00
2820.0	0.88	1.00	0.93
3227.0	1.00	0.86	0.92
3699.0	0.88	1.00	0.93
3745.0	1.00	1.00	1.00
3782.0	1.00	1.00	1.00
4262.0	0.88	1.00	0.93
4740.0	1.00	1.00	1.00
4978.0	1.00	1.00	1.00
6568.0	1.00	1.00	1.00
8968.0	1.00	1.00	1.00
9152.0	1.00	1.00	1.00
9256.0	1.00	0.71	0.83
macro avg	0.97	0.97	0.97
weighted avg	0.97	0.97	0.97
accuracy	0.9693877551020408		
balanced accuracy	0.9693877551020408		

**Table 3:** The effects of PGD eps = 4 on the performance of the classifier.

Label	Precision	Recall	f1-Score	
1757.0	1.00	0.14	0.25
2114.0	0.33	0.14	0.20
2820.0	0.00	0.00	0.00
3227.0	1.00	0.17	0.29
3699.0	0.32	1.00	0.48
3745.0	0.00	0.00	0.00
3782.0	0.00	0.00	0.00
4262.0	0.33	0.71	0.45
4740.0	0.08	0.14	0.11
4978.0	0.00	0.00	0.00
6568.0	1.00	0.14	0.25
8968.0	0.00	0.00	0.00
macro avg	0.40	0.21	0.20
weighted avg	0.38	0.21	0.19
accuracy	0.21052631578947367		
balanced accuracy	0.2139455782312925		

**Table 4:** The results of the classifier using JPEG compression with quality set to 20 on PGD attacks with epsilon = 4.

Label	Precision	Recall	f1-Score	
1757.0	1.00	1.00	1.00
2114.0	1.00	1.00	1.00
2820.0	1.00	1.00	1.00
3227.0	1.00	0.83	0.91
3699.0	0.88	1.00	0.93
3745.0	0.86	0.86	0.86
3782.0	0.86	0.86	0.86
4262.0	0.78	1.00	0.88
4740.0	1.00	1.00	1.00
4978.0	0.86	0.86	0.86
6568.0	1.00	1.00	1.00
8968.0	1.00	0.86	0.92
9152.0	1.00	0.86	0.92
9256.0	0.80	0.80	0.80
macro avg	0.93	0.92	0.92
weighted avg	0.93	0.93	0.93
accuracy	0.9263157894736842		
balanced accuracy	0.9227891156462587		

**Table 5:** The results of the classifier using BMCNN with sigma set to 20 used on adversarial samples created with PGD with epsilon set to four.

Label	Precision	Recall	f1-Score	
1757.0	1.00	1.00	1.00
2114.0	1.00	1.00	1.00
2820.0	1.00	1.00	1.00
3227.0	0.83	0.83	0.83
3699.0	0.70	1.00	0.82
3745.0	1.00	0.71	0.83
3782.0	0.88	1.00	0.93
4262.0	0.78	1.00	0.88
4740.0	1.00	1.00	1.00
4978.0	0.88	1.00	0.93
6568.0	1.00	0.86	0.92
8968.0	1.00	0.86	0.92
9152.0	0.80	0.57	0.67
9256.0	1.00	0.8	0.89
macro avg	0.92	0.90	0.90
weighted avg	0.92	0.91	0.90
accuracy	0.9052631578947369		
balanced accuracy	0.9023809523809525		

**Table 6:** The results of the classifier using spatial smoothing with JPEG compression, Gaussian augmentation, total variance minimisation and BMCNN with sigma set to 20 on PGD images with epsilon set to four.

Label	Precision	Recall	f1-Score	
1757.0	0.50	0.71	0.59
2114.0	0.50	0.43	0.46
2820.0	0.00	0.00	0.00
3227.0	0.40	0.33	0.36
3699.0	0.37	1.00	0.54
3745.0	0.25	0.14	0.18
3782.0	0.25	0.86	0.39
4262.0	0.25	0.14	0.18
4740.0	0.50	0.57	0.53
4978.0	0.67	0.29	0.40
6568.0	1.00	0.14	0.25
8968.0	0.50	0.14	0.22
9152.0	0.67	0.29	0.40
9256.0	0.00	0.00	0.00
macro avg	0.42	0.36	0.32
weighted avg	0.43	0.37	0.33
accuracy	0.3684210526315789		
balanced accuracy	0.36054421768707484		

**Table 7:** The results of the classifier using spatial smoothing with JPEG compression, Gaussian augmentation, and BMCNN with sigma set to 20 on PGD images with epsilon set to four, without total variance minimisation.

Label	Precision	Recall	f1-Score	
1757.0	1.00	1.00	1.00
2114.0	1.00	1.00	1.00
2820.0	1.00	1.00	1.00
3227.0	0.83	0.83	0.83
3699.0	0.78	1.00	0.88
3745.0	1.00	0.86	0.92
3782.0	0.75	0.86	0.80
4262.0	0.78	1.00	0.88
4740.0	1.00	1.00	1.00
4978.0	0.86	0.86	0.86
6568.0	1.00	0.86	0.92
8968.0	1.00	0.86	0.92
9152.0	1.00	0.57	0.73
9256.0	0.83	1.00	0.91
macro avg	0.92	0.91	0.90
weighted avg	0.92	0.91	0.90
accuracy	0.9052631578947369		
balanced accuracy	0.9064625850340137		

**Table 8:** The results of the classifier using spatial smoothing with JPEG compression on PGD images with epsilon set to four.

Label	Precision	Recall	f1-Score	
1757.0	1.00	1.00	1.00
2114.0	1.00	1.00	1.00
2820.0	1.00	1.00	1.00
3227.0	1.00	0.83	0.91
3699.0	0.78	1.00	0.88
3745.0	0.86	0.86	0.86
3782.0	0.86	0.86	0.86
4262.0	0.78	1.00	0.88
4740.0	1.00	1.00	1.00
4978.0	0.86	0.86	0.86
6568.0	1.00	1.00	1.00
8968.0	1.00	0.86	0.92
9152.0	1.00	0.71	0.83
9256.0	0.80	0.80	0.80
macro avg	0.92	0.91	0.91
weighted avg	0.93	0.92	0.92
accuracy	0.9157894736842105		
balanced accuracy	0.9125850340136055		

**Table 9:** The results of the classifier using JPEG compression, Gaussian augmentation, and BMCNN on PGD images with epsilon set to four.

Label	Precision	Recall	f1-Score	
1757.0	0.88	1.00	0.93
2114.0	1.00	1.00	1.00
2820.0	1.00	1.00	1.00
3227.0	1.00	0.83	0.91
3699.0	0.78	1.00	0.88
3745.0	0.86	0.86	0.86
3782.0	0.86	0.86	0.86
4262.0	0.88	1.00	0.93
4740.0	1.00	1.00	1.00
4978.0	0.86	0.86	0.86
6568.0	1.00	1.00	1.00
8968.0	1.00	0.86	0.92
9152.0	1.00	0.71	0.83
9256.0	1.00	1.00	1.00
macro avg	0.94	0.93	0.93
weighted avg	0.93	0.93	0.93
accuracy	0.9263157894736842		
balanced accuracy	0.9268707482993197		

**Table 10:** Results of classification with preprocessing defences on a clean dataset.

Label	Precision	Recall	f1-Score	
1757.0	1.00	1.00	1.00
2114.0	1.00	1.00	1.00
2820.0	1.00	1.00	1.00
3227.0	1.00	0.83	0.91
3699.0	0.88	1.00	0.93
3745.0	0.83	0.71	0.77
3782.0	0.75	0.86	0.80
4262.0	0.78	1.00	0.88
4740.0	1.00	1.00	1.00
4978.0	1.00	1.00	1.00
8968.0	1.00	1.00	1.00
9152.0	1.00	1.00	1.00
9256.0	1.00	0.60	0.75
macro avg	0.95	0.93	0.93
weighted avg	0.94	0.94	0.94
accuracy	0.9368421052631579		
balanced accuracy	0.9289115646258503		

## Data Availability

The dataset used in this study is the Large-Scale CebelFaces Attributes (CelebA) Dataset, available here: https://mmlab.ie.cuhk.edu.hk/projects/CelebA.html (accessed on 30 September 2021).
